# Maternal exposure to ultrafine particles enhances influenza infection during pregnancy

**DOI:** 10.1186/s12989-023-00521-1

**Published:** 2023-04-17

**Authors:** Nicholas L. Drury, Toriq Mustapha, Ross A. Shore, Jiayun Zhao, Gus A. Wright, Aline Rodrigues Hoffmann, Susanne U. Talcott, Annette Regan, Robert M. Tighe, Renyi Zhang, Natalie M. Johnson

**Affiliations:** 1 Department of Environmental and Occupational Health, Texas A&M University, 212 Adriance Lab Rd, 1266 TAMU, College Station, TX 77843 USA; 2 Department of Chemistry, Texas A&M University, College Station, TX 77843 USA; 3 Department of Veterinary Pathobiology, Texas A&M University, College Station, TX 77843 USA; 4 Department of Comparative, Diagnostic, and Population Medicine, University of Florida, Gainesville, FL 32653 USA; 5 Department of Nutrition, Texas A&M University, College Station, TX 77843 USA; 6 School of Nursing and Health Professions, University of San Francisco, Orange County, CA 92868 USA; 7 Department of Medicine, Duke University, Durham, NC 27710 USA; 8 Department of Atmospheric Sciences, Texas A&M University, College Station, TX 77843 USA

**Keywords:** Air pollution, Particulate matter, Ultrafine particles, Pregnancy, Influenza, Infection, Mouse model

## Abstract

**Background:**

Interactions between air pollution and infectious agents are increasingly recognized and critical to identify, especially to protect vulnerable populations. Pregnancy represents a vulnerable period for influenza infection and air pollution exposure, yet interactions during pregnancy remain unclear. Maternal exposure to ultrafine particles (UFPs, $\le$ 100 nm diameter), a class of particulate matter ubiquitous in urban environments, elicits unique pulmonary immune responses. We hypothesized that UFP exposure during pregnancy would lead to aberrant immune responses to influenza enhancing infection severity.

**Results:**

Building from our well-characterized C57Bl/6N mouse model employing daily gestational UFP exposure from gestational day (GD) 0.5–13.5, we carried out a pilot study wherein pregnant dams were subsequently infected with Influenza A/Puerto Rico/8/1934 (PR8) on GD14.5. Findings indicate that PR8 infection caused decreased weight gain in filtered air (FA) and UFP-exposed groups. Co-exposure to UFPs and viral infection led to pronounced elevation in PR8 viral titer and reduced pulmonary inflammation, signifying potential suppression of innate and adaptive immune defenses. Pulmonary expression of the pro-viral factor sphingosine kinase 1 (*Sphk1*) and pro-inflammatory cytokine interleukin-1β (*IL-1* $\beta$) was significantly increased in pregnant mice exposed to UFPs and infected with PR8; expression correlated with higher viral titer.

**Conclusions:**

Results from our model provide initial insight into how maternal UFP exposure during pregnancy enhances respiratory viral infection risk. This model is an important first step in establishing future regulatory and clinical strategies for protecting pregnant women exposed to UFPs.

**Supplementary Information:**

The online version contains supplementary material available at 10.1186/s12989-023-00521-1.

## Introduction

Air pollution is a pervasive environmental health issue worldwide. Globally, air pollution is responsible for one in nine deaths with an annual premature mortality of over 7 million [1–3]. Air pollutants are comprised of a mixture of gases and airborne particulate matter (PM); the latter is typically categorized as ultrafine particles ($\le$ 100 nm in diameter or UFPs), fine particles ($\le$ 2.5 m$\upmu$ or PM_2.5_), and coarse particles ($\le$ 10 m$\upmu$ or PM_10_) [4]. Particle mass concentration is commonly adopted for air pollution control, such as in the U.S. national ambient air quality standards or NAAQS [2]. The particle size largely determines PM deposition patterns along the human respiratory tract [5]. Fine PM has been unequivocally linked to various adverse health effects on humans, ranging from aggravated allergies, development of serious chronic diseases, development of respiratory viral infection, and premature death [1, 3, 6, 7]. PM exposure in vulnerable populations such as pregnant women is especially concerning [5], and gestational exposure to fine PM has been associated with risks for pregnancy-related complications and adverse fetal outcomes [8–12].

Pregnancy is also recognized as a period of vulnerability to severe respiratory infection from multiple viruses, including influenza A virus (IAV), respiratory syncytial virus (RSV), and severe acute respiratory syndrome coronavirus 2 (SARS-CoV-2) [13–15]. Maternal susceptibility to viral infection is explained by several physiological characteristics, including increased cardiac output, and decreased tidal volume, as well as immunological changes such as selective modulation of immune cell subsets to protect the developing fetus [16, 17]. Pregnant women are disproportionately affected by influenza, with a more than ten-fold increase in hospitalization risk even during inter-pandemic periods [16]. Epidemiological evidence from influenza pandemics, including the 2009 influenza A/H1N1 pandemic, reveals increased morbidity and mortality during pregnancy, with the most pronounced risk occurring in the third trimester of pregnancy [13]. Notably, vaccination compliance during pregnancy is generally below 50%, even though maternal vaccination against influenza is safe and effective [18]. Vaccination coverage as of the end of December 2022 was 46.5% for all pregnant persons [19].

**Fig. 1:**
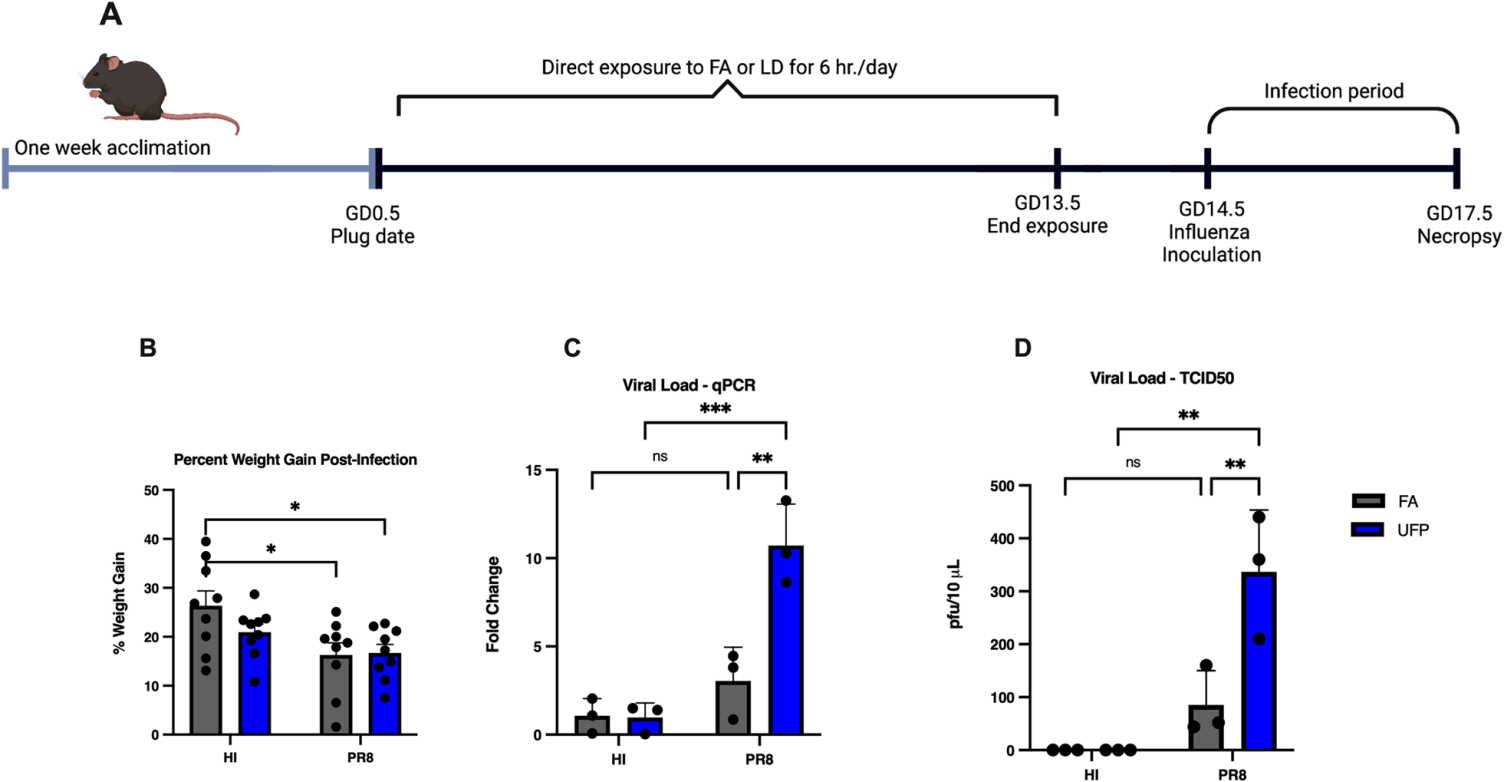
Experimental design and phenotypic outcomes. **A** Experimental timeline indicating the acclimation and mating strategy, exposure duration to FA or UFPs, and viral infection window. **B** Percent weight gain between FA (gray) and UFP (blue) exposure groups post-inoculation. **C** Viral titer assessment via qPCR of the Influenza A segment 7 3’ splice site. **D** Viral titer assessment via TCID50. Data were analyzed using two-way ANOVA with Tukey’s multiple comparison test (*p < 0.05; **p < 0.002; ***p < 0.0002)

Several key aspects of viral respiratory infection during pregnancy remain uncertain, including synergetic effects between gestational PM exposure and respiratory viruses to exacerbate the infectivity, severity, and mortality [20, 21]. Moreover, there is a high degree of exposure misclassification for UFPs, which are not routinely measured in standard air quality monitoring [4, 5]. Atmospheric measurements have shown that the urban atmosphere typically contains high concentrations of UFPs (> 10^4^ particles cm^−3^), which are produced from new particle formation, as well as from emissions of traffic and industrial sources [4, 22–24]. Because of their ability to enter extra-pulmonary tissues and cause systemic oxidative stress, UFPs exhibit significant adverse human health effects [5, 9–11, 25–27]. While PM_2.5_ and PM_10_ are classified as criteria air pollutants under the NAAQS, UFPs are unregulated due to their negligible contribution to the PM mass concentration, and hence the hazards associated with exposure are not fully classified [2, 4, 5]. In this study, we employed a whole-body UFP inhalation model to assess the impacts of UFP exposure during pregnancy (Fig. 1A). The window of exposure (gestational day (GD) 0.5 to 13.5) was based on our previous studies showing effects on fetal growth and offspring respiratory responses [11, 12, 28] with modifications for maternal influenza infection during a susceptible period (i.e., later in pregnancy when severe morbidity from infection is pronounced) [13].The susceptibility of influenza infection from gestational exposure to UFPs was evaluated by multiple endpoints, including weight gain, viral titer, pulmonary histopathology, and pulmonary expression of pro-viral and pro-inflammatory genes. Viral titer is known to peak between 3 and 5 days post infection, coinciding with peak inflammatory cell infiltrates. Moreover, in our models, parturition typically occurs between GD18 and 19 [29]. To also ensure collection of placental tissues, necropsy was performed on GD17.5, 3 days following inoculation with Influenza A/Puerto Rico/8/1934 (PR8). Collectively, results from this pilot study provide initial mechanistic insight into maternal exposure to UFPs and respiratory infection risk, with regulatory and clinical implications for protecting pregnant women.

## Results

### Reduced weight gain and increased viral titer following gestational UFP exposure and PR8 infection

Time-mated C57Bl/6N pregnant mice (dams) were exposed to filtered air (FA) or UFPs equivalent to a 24-h average of 25 $\mu$ g m^3^ (Additional file 1: Fig. S1) over gestational day (GD) 0.5–13.5. Dams were then inoculated with heat-inactivated (HI) control or live Influenza A/Puerto Rico/8/1934 (PR8) on GD14.5 and assessed three days post-infection on GD17.5 prior to delivery. Pregnancy rates for plug-positive females in all exposure groups was ≥ 60%. Because of similarities in pregnancy rates across groups, exposure was not deemed to affect pregnancy loss. Fetal resorptions were not characterized. Weight gain was compared between groups on GD17.5. Percent weight gain in the FA-HI group did not vary significantly from absolute control mice housed in a separate room (data not shown). Noticeable differences were observed in the percent weight gain for dams exposed to FA or UFP and inoculated with PR8, indicating robust infection (Fig. 1B). The percent weight gain was impacted in both infected groups (*i.e*., FA-HI *vs*. FA-PR8, and FA-HI and UFP-PR8; *n* = 9/group), indicating a successful infection. No differences in percent weight gain was noted between UFP-HI *vs*. UFP-PR8 or FA-PR8 *vs*. UFP-PR8.

To quantify PR8 infectivity, viral titer was determined in the lung using quantitative polymerase chain reaction (qPCR) and the 50% tissue culture infectious dose (TCID50) assay. The qPCR analysis of the influenza A segment 7 3’ splice site encoding the M1 and M2 viral proteins showed the highest expression in the UFP-PR8 group, elevated 3.7-fold compared to the FA-PR8 group (*p* = 0.0027; Fig. 1C). The qPCR results were also corroborated by TCID50 analysis, with a fivefold higher PR8 lung titer in the UFP-PR8 group than the FA-PR8 group (*p* = 0.0075; Fig. 1D). Additionally, TCID50 analysis exhibited extensive PR8 cytopathic effect (CPE) in the UFP exposure group compared to the FA exposure group, reflected by excessive cell rounding, lysis, and multifocal cellular destruction. Together, the qPCR and TCID50 results of PR8 lung titer indicated that UFP exposure increased viral replication and infectivity.

### Impaired pulmonary immune responses to PR8 infection

**Fig. 2:**
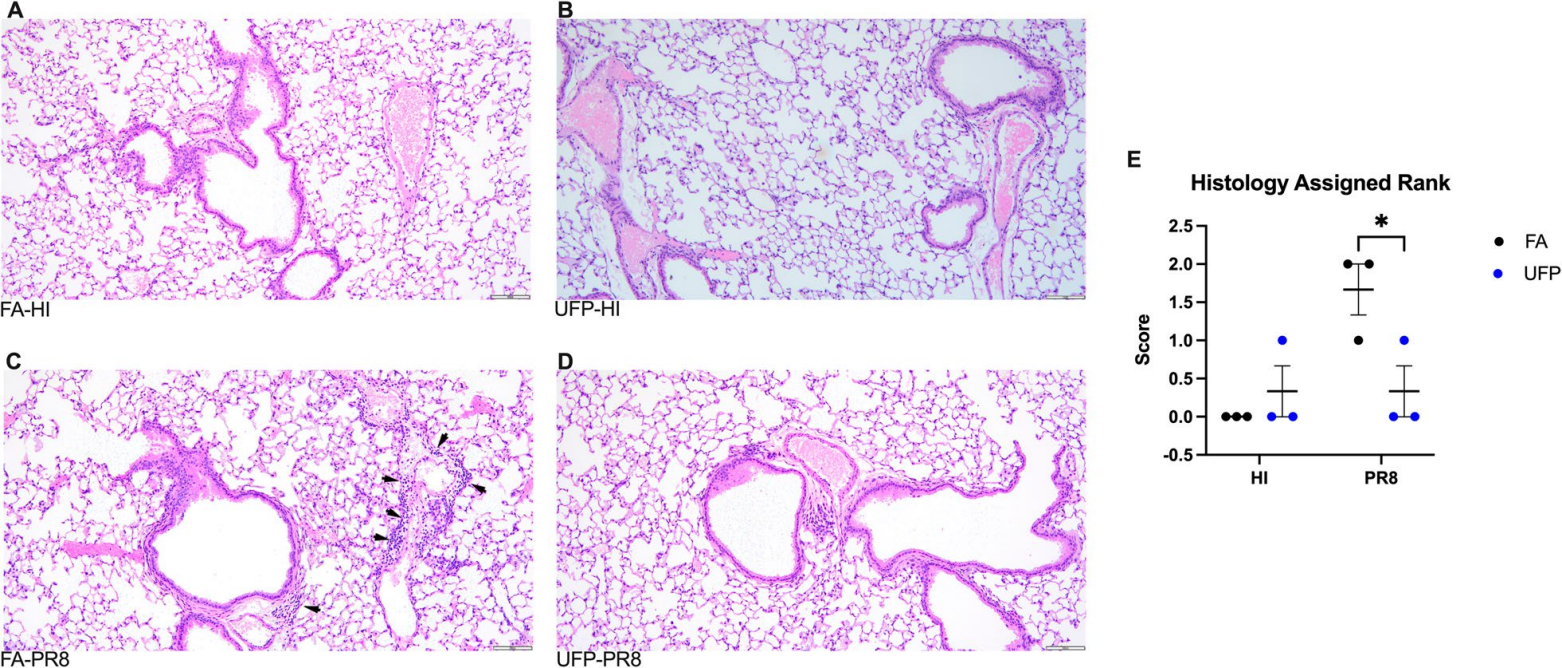
Pulmonary histopathology. Two histological slides from a longitudinal section from the lungs including the mainstem bronchi and both right and left side lung lobes were evaluated for each animal. Representative area of average scores is shown (photographed at 20X magnification). **A** Dam exposed to FA and inoculation with HI, showing no histological lesions (avg. score = 0). **B** Dam exposed to PM and inoculated with HI, showing no histological lesions (avg. score = 0.33). **C** Dam exposed to FA and inoculated with PR8, showing moderate perivascular and peribronchiolar lymphoplasmacytic, histolytic and neutrophilic inflammation denoted by arrows (avg. score = 1.66). **D** Dam exposed to PM and inoculated with live PR8, showing minimal numbers of peribronchial lymphocytes (avg. score = 0). **E** Pathological scoring of lungs in each exposure group

We evaluated pulmonary histopathology three days post-infection using a semi-quantitative scoring system. The most severe case in each group is shown (Fig. 2). Overall, dams in the FA-HI and UFP-HI groups showed no significant inflammatory infiltration, except for one dam in the UFP-HI group which had mild and focal inflammation (Fig. 2A and B). Generally, inflammation was more apparent in the FA-PR8 group, (Fig. 2C) which consisted of mild to moderate and mostly focal to scattered infiltrates of lymphocytes, macrophages, plasma cells and scattered neutrophils, scattered confined to peribronchial and perivascular regions, with no involvement of major airways and alveolar spaces. Similar to the HI groups, the UFP-PR8 group did not present significant inflammation, other than scattered minimal aggregates of lymphocytes (score = 0). The absence of inflammatory infiltrates in the UFP-PR8 group, in contrast to FA exposure, suggests innate immune suppression. In clinical cases of severe influenza infection, commonly observed histopathological lesions include extensive innate immune cell infiltration (especially neutrophils), damage to the epithelial lining to cause edema and hyaline membrane formation, and alveolar collapse, which collectively induce acute respiratory distress syndrome characterized by diffuse alveolar damage and respiratory failure [30–32]. In addition, PM causes immunosuppression through stimulation of IL-10 signaling, reducing innate immune defenses, and reduction in inflammatory signaling during pregnancy also results in immune system tolerance [33, 34].

**Fig. 3:**
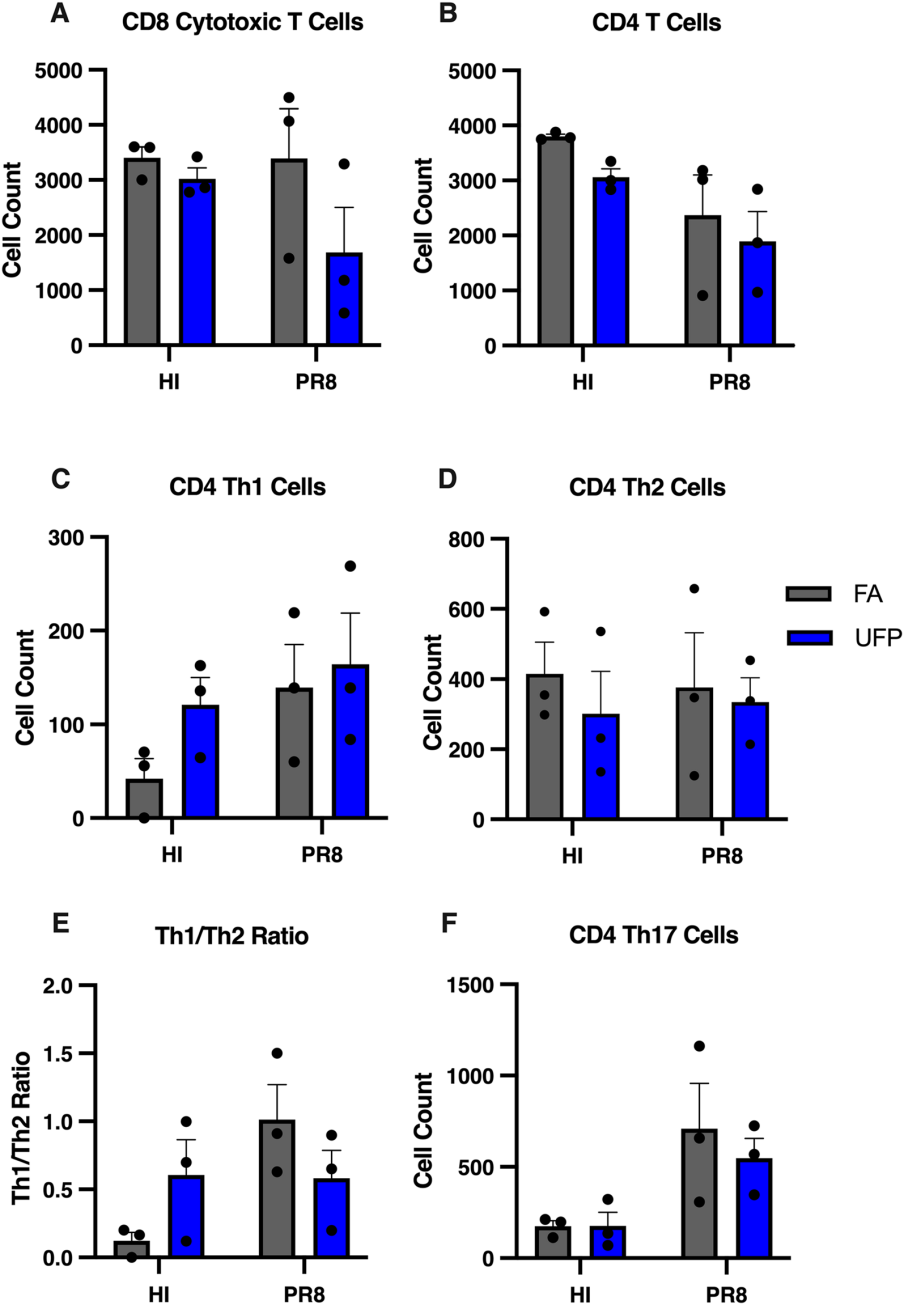
Pulmonary T cell profiles from dissociated lung tissue. **A** CD8 + T cell counts, showing noticeable reduction in the UFP-PR8 group compared to all other groups. **B** CD4 + T cell counts. **C** Count of CD4 + T cells belonging to the Th1 lineage (IFN- +). **D** Count of CD4 + T cells belonging to the Th2 lineage (IL-4 +). **E** Th1/Th2 cell ratio in the lung, used as an indicator of immune system bias. **F** Count of CD4 + T cells belonging to the Th17 lineage (IL-17 +), showing a slight reduction in the UFP-PR8 group compared to the FA-PR8 group

We analyzed the pulmonary T cell responses in from homogenized maternal lung samples in each exposure group, including the subsets Th1, Th2, Th17, and CD8 + cytotoxic T lymphocyte (CTL) lineages. The exposed and inoculated groups revealed shifted immune cell profiles suggestive of pulmonary immunosuppression (Fig. 3A–F). The UFP-PR8 group showed the lowest levels of CD8 + cytotoxic lymphocytes. The CD8 + T cells are capable of rapidly expanding and identifying infected host cells for programmed cell death [35, 36]. The reduced CD8 + T cell population in the UFP-PR8 group provided additional evidence of early immune suppression, even in the adaptive arm, consistent with the elevated PR8 lung titer and reduced tissue pathology (Figs. 1 and 2). In our study, the lack of significant differences between T cell subsets may be explainable by the narrow infection window, which we selected to evaluate prior to delivery. The flow cytometry data are more variable and appear to require additional investigation to draw affirmative conclusions. T cell kinetics to influenza infection has been described for both mice and humans [37, 38]. Typically, IAV-specific CD8 + T cells remain at the baseline level from 0 to 2 days post-infection, followed by a single proliferation cycle in the lung by 4 days post-infection and subsequent rapid expansion from days 5–9 post-infection. Similarly, CD4 + T cell responses do not reach their peak until days 7–9 post-infection.

### Dynamic changes in pro-viral and pro-inflammatory gene expression

**Fig. 4:**
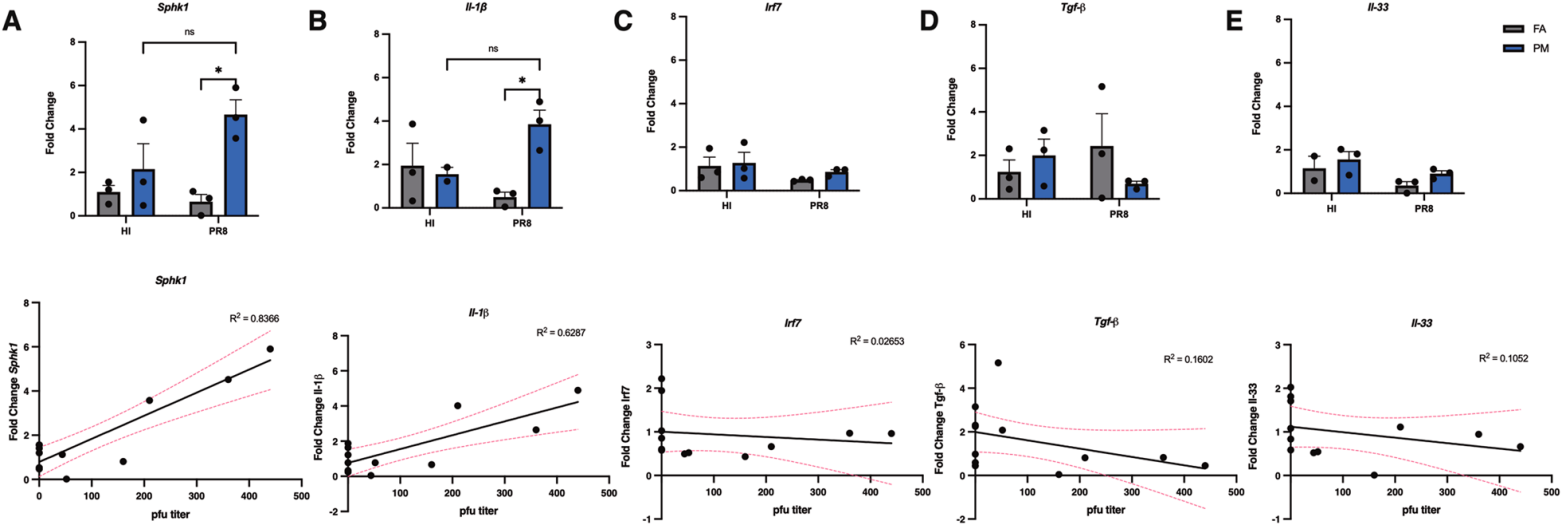
Regulation of pro-viral and pro-inflammatory genes **A** Expression of *Sphk1* between exposure groups, with the highest expression UFP-PR8 group; correlation analysis showed a strong positive correlation with viral titer (R^2^ = 0.8366). **B** Expression of *Il-1β* between exposure groups, with the highest expression in the UFP-PR8 group; correlation analysis showed a moderate positive correlation with viral titer (R^2^ = 0.6287). **C** Expression of *Irf7* between exposure groups, with no significant changes or correlations with viral titer (R^2^ = 0.02653). **D** Expression of *Tgf-β* between exposure groups with no significant changes or correlations (R^2^ = 0.1602). **E** Expression of *Il-33* between groups with no significant changes or correlations with viral titer (R^2^ = 0.1052)

We examined several genes via qPCR in the lungs including transcription factors, genes encoding for inflammatory cytokines, and immune-cell specific genes (Fig. 4). These transcription factors, including the *Sphk1* gene and *Il-*$\beta$ cytokine gene, play an important role in the induction of myeloid cells in the innate immune response. Detectable expression was observed in five genes (Fig. 4A–E). A moderately strong positive correlation with PR8 lung titer was identified for *Sphk1* (Fig. 4A and *Il-1β* (Fig. 4B); there was little correlation for *Irf7*, *Tgf-β*, and *Il-33* (Fig. 4C–E). The increased expression for *Sphk1* and *Il-1β* was consistent with an increase in viral titer (Fig. 1C–D). *Sphk1* showed an approximate fivefold increase in the UFP-PR8 group compared to the FA-PR8 group (*p* = 0.0171; Fig. 4A). *Sphk1* is a gene encoding for a kinase that controls cellular differentiation and apoptosis and serves as an important pro-viral factor by regulating the synthesis of viral RNA and export of the viral ribonucleoprotein complex upon infection with influenza. In cases of severe influenza infection, *Sphk1* level and activity have been shown to be increased, promoting replication of influenza. *Il-1* $\beta$ showed a significant increase in expression in the UFP-PR8 group compared to the FA-PR8 group (*p* = 0.0377; Fig. 4B). *Il-1* $\beta$ is a key mediator of the inflammatory response, essential for host response and resistance to pathogens [39–41]. Despite the high expression of this pro-inflammatory signaling cytokine, evidence for pulmonary inflammation is absent, which suggested the expression is not signaling immune effector cells important for early resolution of PR8.

Considering the susceptibility to infection during pregnancy, transcriptional analysis of several genes in the placenta was performed to provide additional context to the systemic factors that could contribute to pulmonary susceptibility. We assessed expression of matrix metalloproteinase 9 (*Mmp9*), cyclooxygenase 2 (*Cox-2*), placenta-derived growth factor (*P1gf*), prostagalandin E synthase 2 (*Pges2*), progesterone induced blocking factor (*Pibf*), and vascular endothelial growth factor (*Vegf*) and. *Mmp9* cleaves various proteins to regulate the response to inflammation and injury, and increased *Mmp* 9 expression has been found to have a positive association with H1N1 viral titer [42]. *Cox-2* is an immune-responsive hormone regulator, shown to be dysregulated in H1N1-infected pregnant mice [43]. *Pges2* catalyzes the synthesis of prostaglandin E_2_ (PGE_2_), an important lipid mediator with evidence for suppressing the innate immune response to IAV; *Pibf is a progesterone-sensitive immunomodulatory gene promoting Th2 cytokine production and inhibition of NK cells* [44]. *Vegf* regulates vascular, inflammation, remodeling, and cell-death, contributing to vascular changes in the placenta but also roles in pulmonary physiology [45]. Although five of the genes showed detectable expression, no significant differences in gene expression were observed between placentas of either sex for any exposure group (Additional file 1: Fig. S4).

### Maternal infection did not affect fetal measures.

We examined endpoints from each fetus from all exposure groups, including fetal weight, fetal crown-to-rump length, placental weight, and placental efficiency (Additional file 1: Fig. S3). With the exception of a downward trend in male and female placental efficiencies in the UFP-PR8 exposure group, no discernible pattern in fetal measures was noted, and no changes were statistically significant.

## Discussion

**Fig. 5:**
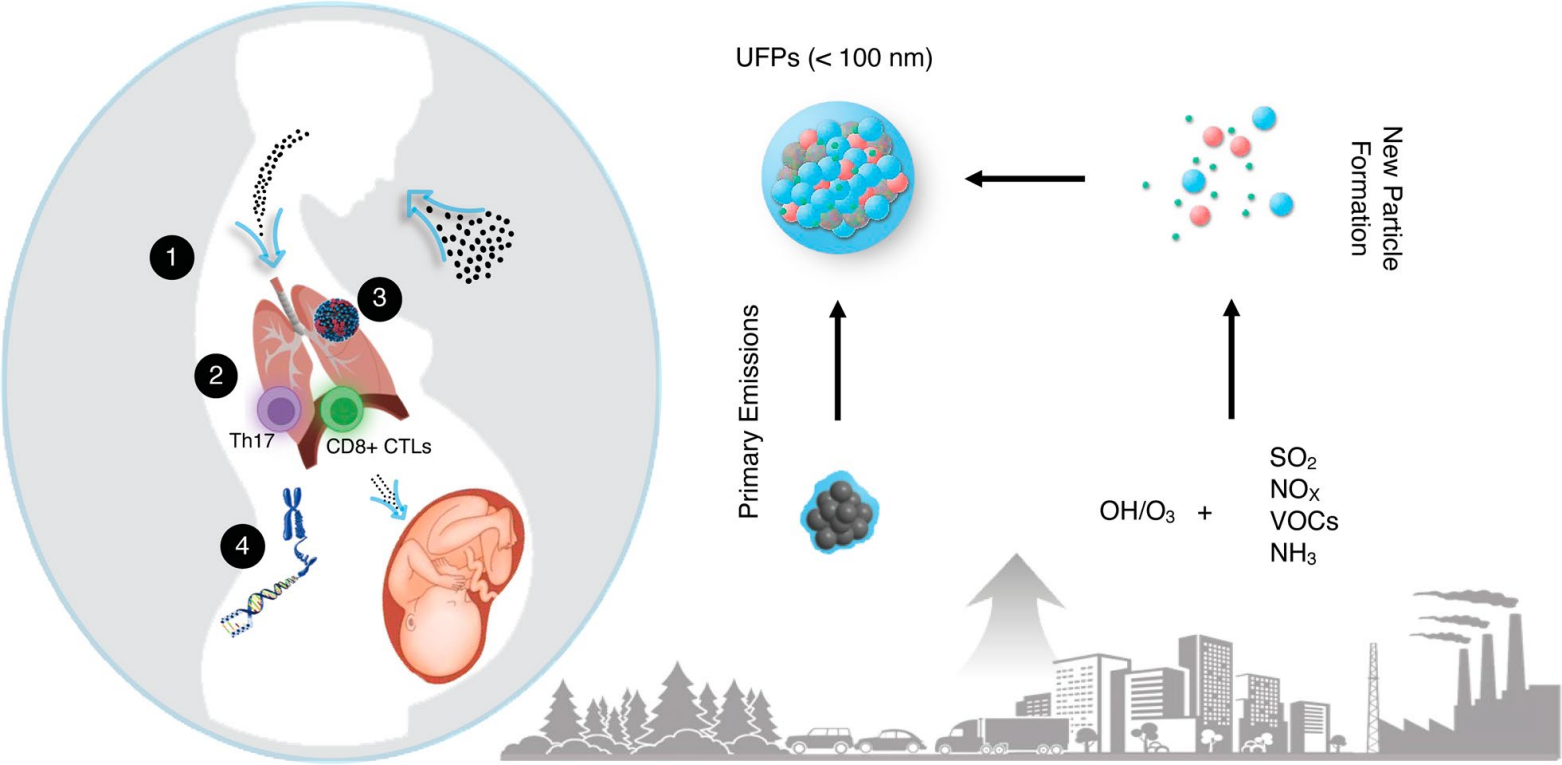
Schematic depicting the sources of UFPs and the effects on influenza infection severity observed in our model. In urban environments, high number concentrations of UFPs are directly emitted into the atmosphere from traffic and industrial sources and/or produced from new particle formation relevant to photochemical oxidation, which is initiated by the hydroxyl radical (OH) or ozone (O_3_), involving volatile organic compounds (VOCs) and sulfur dioxide (SO_2_) in the presence of nitrogen oxides (NO_x_ = NO + NO_2_) and ammonia (NH_3_)^4,20–22^. Our mouse model illustrates four major adverse health effects for aggravated respiratory infection from UFP exposure for pregnant women: (1) reduced weight gain, (2) reduced pulmonary immune responses, (3) elevated viral titer, and (4) enhanced pro-viral and pro-inflammatory gene expression

The factors governing the interactions between air pollution and severity of respiratory viral infection are complex and multifactorial, combining aspects of toxicology, immunology, and individual biology. These interactions may also depend on the deposition characteristics and chemistry of the particles inhaled. Because of their high abundance in urban environments and ability to penetrate deeper into the human bodies and cause systemic oxidative stress [4, 5, 21–23], UFPs likely exert profound adverse health effects on pregnant women (Fig. 5). Previous studies demonstrate that prenatal exposure to UFPs is associated with stillbirth, low birth weight, and adverse organogenesis as well as respiratory, cognitive, and cardiometabolic dysfunctions relevant to both conceptus and postnatal growth and development [9–11]. Our mouse model provides multiple lines of supporting evidence for enhanced susceptibility to influenza infection from gestational UFP exposure, demonstrating reduced weight gain (Fig. 1B), elevated viral titer (Fig. 1C–D), reduced inflammation in lungs and T cell response (Figs. 2 and 3), and downregulated inflammatory mediators and upregulated pro-viral factors (Fig. 4). While *Il-1β*, a pro-inflammatory mediator, is elevated in the UFP-PR8 group, antiviral inflammation is largely suppressed, based on the absence of inflammatory cell infiltrates in the lungs. Absence of the inflammatory cell infiltrates critical for early resolution of infection demonstrates the absence of inflammatory chemotactic signaling, despite elevated *Il-1β*.

The weight gain serves as a key marker for healthy pregnancy progression and in viral models as an indicator for infection severity. Inadequate weight gain is associated with increased risks for infant death, failure to initiate breastfeeding, and preterm birth [46]. Innate immune effector cells, including neutrophils, macrophages, and eosinophils, characteristic of the first line defenders against invading pathogens are largely absent in histopathological cases for the UFP-PR8 group. The innate immune cells also serve to signal the adaptive arm of the immune system, dominated by the T helper cells and CD8 + CTLs. Specifically, neutrophils lay down tracks in the trachea upon infection to guide CD8 + CTLs, and ineffective signaling causes defective CD8 + T cell recruitment [46]. Both innate and adaptive immune cell subsets, especially CD8 + CTLs, are integral in effective viral clearance, and a reduction in activity corresponds to reduced antiviral activity and host defense [47]. In our pilot study, analysis of homogenized lung samples for Th1, Th2, Th17, and CD8 + T cells revealed shifted immune cell profiles. Hence, the increased viral titer corroborates reduced early innate and adaptive immune response in the lungs. Additionally, maternal immune system modulation during pregnancy may affect the response to viral infections. The altered inflammatory response is characterized by a shift in the CD4 + T cell population toward the Th2 immunophenotype over the Th1 immunophenotype (the Th1/Th2 "shift"). Decreased Th1 reactivity can affect the clearance of infected cells. Increases in progesterone levels has been shown to decrease virus-specific antibody levels and influenza-specific CD8 + T-cells. Other changes in immune function, including decreased circulating natural killer (NK) cells, are also implied in increased host susceptibility during pregnancy. This underscores the importance of investigating a comprehensive immune system panel for fully characterizing infection severity and risk [14].

Significantly elevated *Sphk1* and *Il-1β* in the UFP-PR8 group are positively correlated with viral titer and infection severity. Interestingly, Vose et al. observed a reduction in *Sphk1* transcript levels, correlating with the reduced viral titer observed in non-pregnant mice exposed to wood smoke particles (WSP) and subsequently infected with PR8. It was suggested that the WSP could reduce *Sphk1* levels as a downstream effect, but this was not conclusively determined. Differences in PM composition or exposure factors, namely pregnancy, may explain these differing results [48]. Prior studies demonstrate differential responses to various particles in pregnant mice. For instance, Fedulov et al. showed that pregnancy enhanced the inflammatory response to TiO_2_ particles, in comparison to non-pregnant mice [49]. Conversely, Thaver et al. demonstrated pregnancy reduced the inflammatory response to silica and altered the immune response to diesel exhaust PM, increasing the proportion of CD4^+^CD25^+^T cells in response to PM exposure [50]. These findings underscore the importance of characterizing particle size and composition, to investigate the unique effects during pregnancy. Considering high susceptibility to infection during pregnancy, transcriptional analysis of several genes in the placenta was performed to provide additional context to systemic factors that could contribute to pulmonary susceptibility. No significant increase or decrease was observed in any of the examined genes, despite their known correlation with influenza infection. We attribute this to the narrow infection window utilized for the study.

Our model is a useful start to tease apart effects of UFPs during pregnancy. Our study does have some limitations. First, our model investigates only a single dosage level of UFPs, at 25 g$\mu$/m^3^ over a 24-h period. This dosage level is below the EPA standards for PM_2.5_, the guideline for which is a 24-h with 98^th^ percentile forms and levels of 35 g$\mu$/m^3^, serving as the primary and secondary standard [51]. Our study utilized a dose of 25 μg/m^3^ UFPs for 6 h for 13 days, followed by a three-day infection challenge. While our dose does approach a regulatory standard, the addition of an alternative dosing level surpassing the regulatory standard is warranted to determine a dose–response relationship between UFPs and PR8 viral infection. Second, our infection window is relatively narrow. A narrow 3-day infection window was selected on the basis that it would be an adequate duration for innate immune activation, and ensure tissue collection prior to spontaneous parturition at GD18.5 or GD19.5However, the restrictive window likely explains the lack of more substantial histopathological lesions amongst exposure and inoculum groups. Consistent with this observation is the lack of significant changes in T cell profiles between groups, which requires further investigation. Innate immune effector cells, including macrophages, neutrophils, NK cells, and dendritic cells, were not examined. In future models expanding on this work, a comprehensive flow cytometry panel is required. Additionally, maternal influenza infection is documented to contribute to adverse pregnancy outcomes and pro-abortive mechanisms through tissue-specific hormonal dysregulation, which was not apparent in our study. Therefore, future models extending the infection period are warranted.

Collectively, our results are an important start to support clinical and regulatory interventions for protecting pregnant women and controlling UFPs, respectively. It is imperative to provide vaccination for pregnant women in urban cities, where influenza and UFPs have common hot spots. Moreover, low-income and minority populations are more likely to live closer to traffic and industrial-related UFP sources, and the combined vulnerabilities of pollution exposure, race, and social factors pose a major challenge regarding environmental justice and equity [52]. Preventive measures limiting UFP exposure and promoting vaccination are warranted to protect maternal health.

## Methods

### Animals and exposures

#### Breeding

Male and female C57Bl/6N 8- to 10- week-old mice (Jackson Laboratory, Bar Harbor, Maine) were kept in a 12-h light dark cycle and time mated in an AAALAC approved facility at Texas A&M Institute for Genomic Medicine. All approved protocols were followed according to the Texas A&M University Institutional Animal Care and Use Committee #2019–0025. Female mice were housed two per cage in polycarbonate cages with corncob bedding in controlled conditions (12/12 h light/dark cycle, 18–23 C, 50% humidity). Male breeders were housed one per cage in a similar manner. Mice had access to standard chow, 19% protein extruded diet (9% fat) and water *ad lib* except during exposure periods*. *Following a two-week acclimation period to the housing room, female mice were acclimated to the inhalation exposure chambers for 6 h/day for 1 week to reduce the potential for exposure-related pregnancy loss due to stress. Females were also acclimated to vaginal cytology for an additional two days prior to time mating. To determine estrus cycle phase, 20 $\mu$ L of PBS was injected into the vagina, quickly aspirated, and the aspirate examined to assess the cells present. Females determined to be in either proestrus or estrus were separated for mating. In the evening, females were co-housed with males, and the presence of a copulatory plug the following morning was designated gestational day (GD) 0.5. Initial weights were recorded, and dams were randomly assigned to filtered air (FA) control or PM exposure groups. Maternal weights were assessed daily throughout exposure and infection.

#### Ultrafine particulate matter exposures

PM exposure was carried out as previously described in Behlen et al. and Lau et al. [12, 27, 53]. Mice (*n* = 18 for FA; *n* = 18 for UFPs) were exposed from GD0.5 to 13.5 for 6 h/day in whole-body inhalation chambers. Briefly, HEPA-filtered air was continuously pumped into FA and UFP chambers. Noise levels from pump operation were found to be negligible. The UFP aerosol, consisting of ammonium nitrate, ammonium sulfate, diesel soot (NIST, SRM 2975) and potassium chloride was generated with a constant output atomizer. Particle size distribution and count was monitored in real-time using a differential mobility analyzer (DMA) and condensation particle counter (CPC). The UFP chamber was monitored, and the flow of HEPA-filtered air adjusted accordingly to maintain particle concentrations of ~ 100 g$\mu$/m^3^ throughout the study. This dose was based on previously observed effects on offspring health [12, 27, 53]. Moreover, this dose was equivalent to a 24-h average exposure 25 g$\mu$/m^3^, just under the EPA PM_2.5_ regulatory level (35 g$\mu$/m^3^). Dams judged not to be pregnant based on weight gain at the conclusion of the exposure duration were either returned to the breeding pool (FA exposed) or euthanized (UFP exposed). Dams judged to be pregnant were transferred to an ABSL2 suite for infection with influenza.

#### Viral challenge

Following exposure to FA or UFPs, dams acclimated overnight at the Texas A&M Laboratory Animal Care Facility. The following day, dams were placed under light isoflurane anesthesia and inoculated with 6.2 × 10^4^ pfu of live Influenza A/Puerto Rico/8/1934 (PR8) or heat-inactivated (HI) control in a total volume of 20 mL via intranasal administration. This gave four total exposure groups: FA-HI, FA-PR8, UFP-HI, and UFP-PR8 (*n* = 9/group). The dose was selected to model severe cases of human influenza infection. PR8 was provided by Dr. Robert Tighe (Duke University), and viral stocks and controls were generated as previously described [54, 55]. Dams were monitored for any signs of distress, including dyspnea, hunched posture, orbital tightening, and excessive weight loss. Three days post-infection (3 dpi), dams were allocated to one of three necropsy groups: viral load and antiviral qPCR (lungs snap frozen in liquid nitrogen), histopathology (lungs inflated at a constant pressure of 25 cm and fixed with zinc formalin), or flow cytometry (lungs processed into single cell suspensions) [28]. Females were euthanized by intraperitoneal injection of 200 mg/kg pentobarbital.

Following removal of the lungs, the abdomen and peritoneum of each pregnant dam were opened, and the abdominal organs were moved aside to visualize the uterus and the ovaries. The ovaries distal to the oviduct and uterotubal junction of each horn were located, and an incision made at the uterotubal junction to separate the uterine horns from the mesentery containing the uterine vessels. To isolate each conceptus, transverse cuts were made through the inter-implantation regions. The placental and decidual tissues were removed from each fetus. Crown-to-rump length (CRL), fetal weight, and placental weight were determined and sex-separated. Placentas were snap-frozen until used for transcriptional analyses.

### Pulmonary histopathology

Lungs (*n* = 3/group) were fixed in formalin for 24–48 h. Tissues were transversely trimmed and transferred to 70% ethanol for storage until embedding and staining with hematoxylin and eosin (H&E) to identify cellular infiltrates. All histological assessments were carried out by an anatomic pathologist blinded to treatment groups. A scoring system was used as follows to rate inflammation severity: 0 (none to minimal), 1 (mild), 2 (moderate), and 3 (marked).

### Pulmonary flow cytometry

Lungs (*n* = 3/group) were perfused with sterile PBS to deplete red blood cells and processed into single cell suspensions for flow cytometry as previously described [27]. Cells were stained with antibodies for CD3, CD8, CD4, IFN-*γ*, IL-4 and IL-17 to determine CD8 + and CD4 + Th1/Th2/Th17 responses, respectively. T cell staining used monoclonal Alexa Fluor (AF) 488 anti-mouse CD3, and PE anti-mouse CD69. Th1 staining used monoclonal AF 647 IFN-γ, APC/Fire 750 anti-mouse CD4, and PE/Cy7 anti-mouse CD8a. Th2 staining used monoclonal BV421 anti-mouse IL-4. Th17 staining utilized monoclonal AF 488 anti-mouse IL-17A. Immunostained cells (10,000/sample) were analyzed on a Luminex/Amnis Cell Stream flow cytometer as previously described [27]. The flow cytometry data was analyzed using Cell Stream analysis software (Luminex/Amnis). Flow cytometry gating strategy reported in Additional file 1: Fig S2.

### Pulmonary viral load

Lung samples collected for viral load determination (*n* = 3/group) were assessed via the TCID50 assay or qPCR. For the TCID50 assay, confluent 96 well plates of MDCK cells (ATCC, CCL-34) were cultured in standard DMEM culture media, supplemented with 10% FBS, 1% antibiotic and antimycotic, L-glutamine, and 3.7 g/L of sodium bicarbonate. Lung samples were homogenized in PR8 influenza infection medium, as described previously [56]. The first column of 96-well plates of MDCK cells were inoculated with 10 $\mu$ L of lung homogenate, while the additional columns were inoculated with serial 10X dilutions of the homogenate. The final well volume was 200 $\mu$ L. The plate was incubated for 5 days or until cytopathic effect (CPE) was evident in all wells of the first column of the plate. For qPCR, RNA was isolated from lung samples using the TRIzol method. RNA purity was determined following the isolation step. Samples with RNA quantities $\le$ 200 ng/$\mu$ L or low A260/A280 ratios ($\le$ 1.6) were rejected, and new RNA samples were prepared. Complementary DNA was synthesized from 1 µg of total RNA (Qiagen QuantiTect^®^ Reverse Transcription Kit, Cat #205,311), and qPCR was conducted utilizing the PowerSYBR Green PCR Master Mix (Applied Biosystems, Cat #4,367,659) on a Roche LightCycler^®^ 96 PCR machine. Master mixes were prepared, including primers for the PR8 segment 7 3’ splice site gene. *Gapdh* was used as a reference gene. No template controls (NTCs) and no reverse transcription controls (−RT) were used as negative controls for all genes and samples. Relative gene expression was determined using the ${2}^{-\Delta \Delta Ct}$ method [57].

### Antiviral gene expression analysis

Lung samples for viral load determinations (*n* = 3/group) were also used for an antiviral qPCR assessment. To determine the impact of PM on the antiviral response against influenza PR8 infection, an additional panel of genes integral to the antiviral response were assessed. Primers for each gene are reported in Additional file 1: Table S1. The genes analyzed included those encoding common antiviral cytokines (*Tgf-*$\beta$*, Il-1 *$\beta$*, Il-33*) involved in chemotactic signaling and inflammatory response, and upstream transcription factors (*Irf5, Irf7, Stat4, Sphk1*) involved in regulation of the interferon responses and viral progeny production. *Nos2*, a marker for classically activated macrophages, was also evaluated. Relative gene expression between groups was determined using the ${2}^{-\Delta \Delta Ct}$ method, normalizing to the FA-HI control [57]. Primer sequences are reported in Additional file 1: Table S1.

### Placental gene expression analysis

To determine the impact of UFPs on cytokines and hormones in the placenta that may contribute to pulmonary susceptibility to influenza, placentas were collected upon necropsy for all positive pregnancies, weighed, and sex-separated. Placentas were pooled within litters (*n* = 3 placentas per sex, per exposure group; *n* = 3 litters per exposure group). The genes analyzed included *Mmp9*, enzyme-coding genes (*Pges2*, *Cox-2*), and growth factors (*P1gf* and *Vegf*). Primer sequences are reported in Additional file 1: Table S2.

### Statistical analysis

All statistical analyses were performed using GraphPad Prism (V 9.2.0) and expressed as mean ± SEM. Phenotypic analyses were tested using two-way analysis of variance (ANOVA) with Tukey’s multiple comparisons test. Tests were considered statistically significant with a *p* value < 0.05.

## Supplementary Information

**Additional file 1.** Supplementary Figures and Tables.

## Data Availability

The datasets used and/or analyzed during the current study are available from the corresponding author on reasonable request.
