# Supplementary material for: Maternal exposure to ultrafine particles enhances influenza infection during pregnancy
Source: Part Fibre Toxicol. 2023 Apr 17;20:11. doi: 10.1186/s12989-023-00521-1 (PMC10106898; doi:10.1186/s12989-023-00521-1)
Supplement: Supplementary file 1 — Additional file 1. Supplementary Figures and Tables. [file 12989_2023_521_MOESM1_ESM.docx]

Supplementary Materials for

Maternal Exposure to Ultrafine Particles Enhances Influenza Infection During Pregnancy

**Authors:** *Nicholas L. Drury^1,5^*; Toriq Mustapha^1^; Ross A. Shore^1^; Jiayun Zhao^2^; Gus A. Wright^3^; Aline Rodrigues Hoffmann^4^; Susanne U. Talcott^5^; Annette Regan^6^; Robert M. Tighe^7^; Renyi Zhang^2,8^; Natalie M. Johnson^1^*

^1^ Department of Environmental and Occupational Health, Texas A&M University, College Station, TX 77843.

^2^ Department of Chemistry, Texas A&M University, College Station, TX 77843.

^3^ Department of Veterinary Pathobiology, Texas A&M University, College Station, TX 77843.

^4^ Department of Comparative, Diagnostic, and Population Medicine, University of Florida, Gainesville, FL 32653.

^5^ Department of Nutrition, Texas A&M University, College Station, TX 77843.

^6^ School of Nursing and Health Professions, University of San Francisco, Orange County, CA 92868.

^7^ Department of Medicine, Duke University, Durham, NC 27710.

^8^Department of Atmospheric Sciences, Texas A&M University, College Station, TX 77843.

*Correspondence should be addressed to: Natalie Johnson, Email: [nmjohnson@tamu.edu](mailto:nmjohnson@tamu.edu). Address: 212 Adriance Lab Rd, 1266 TAMU, College Station TX 77843.

This PDF file contains:

Figure S1

Figure S2

Figure S3

Figure S4

Table S1

Table S2


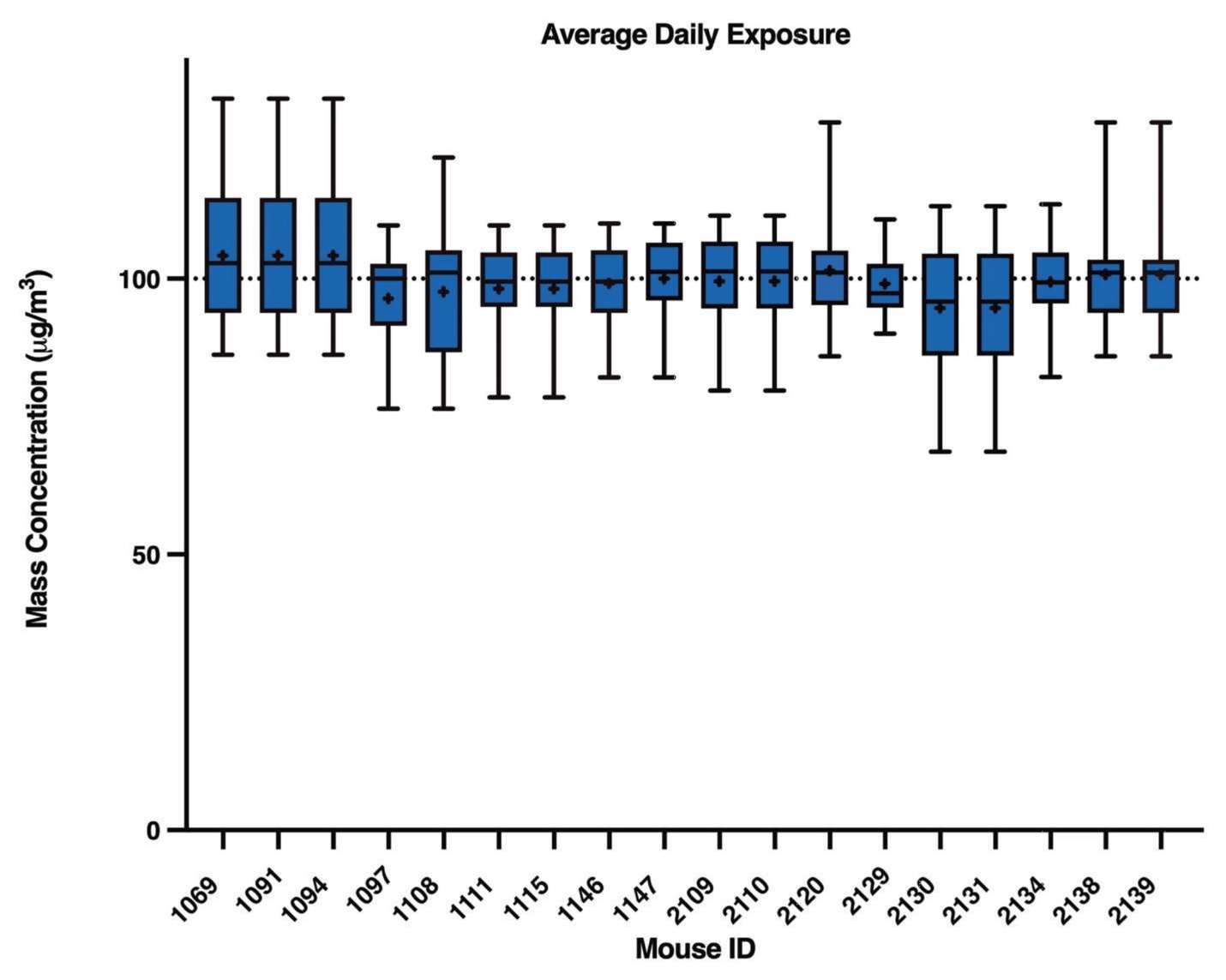


**Fig. S1**

**Average daily PM exposure.** The average daily exposure equaled 99.56 $\boldsymbol{\pm}$ 2.83 $\boldsymbol{\mu}$g/m^3^. The average exposure for each dam is indicated as a "+" mark on each bar. The target dose (100 $\boldsymbol{\mu}$g/m^3^) is outlined, as shown by dotted line. Dams starting with "1" in their ID were subsequently inoculated with live PR8, and dams starting with “2” in their ID were inoculated with heat-inactivated PR8 (HI). Exposure was carried out over the same time period for both groups, and no differences in exposure levels were observed between groups.


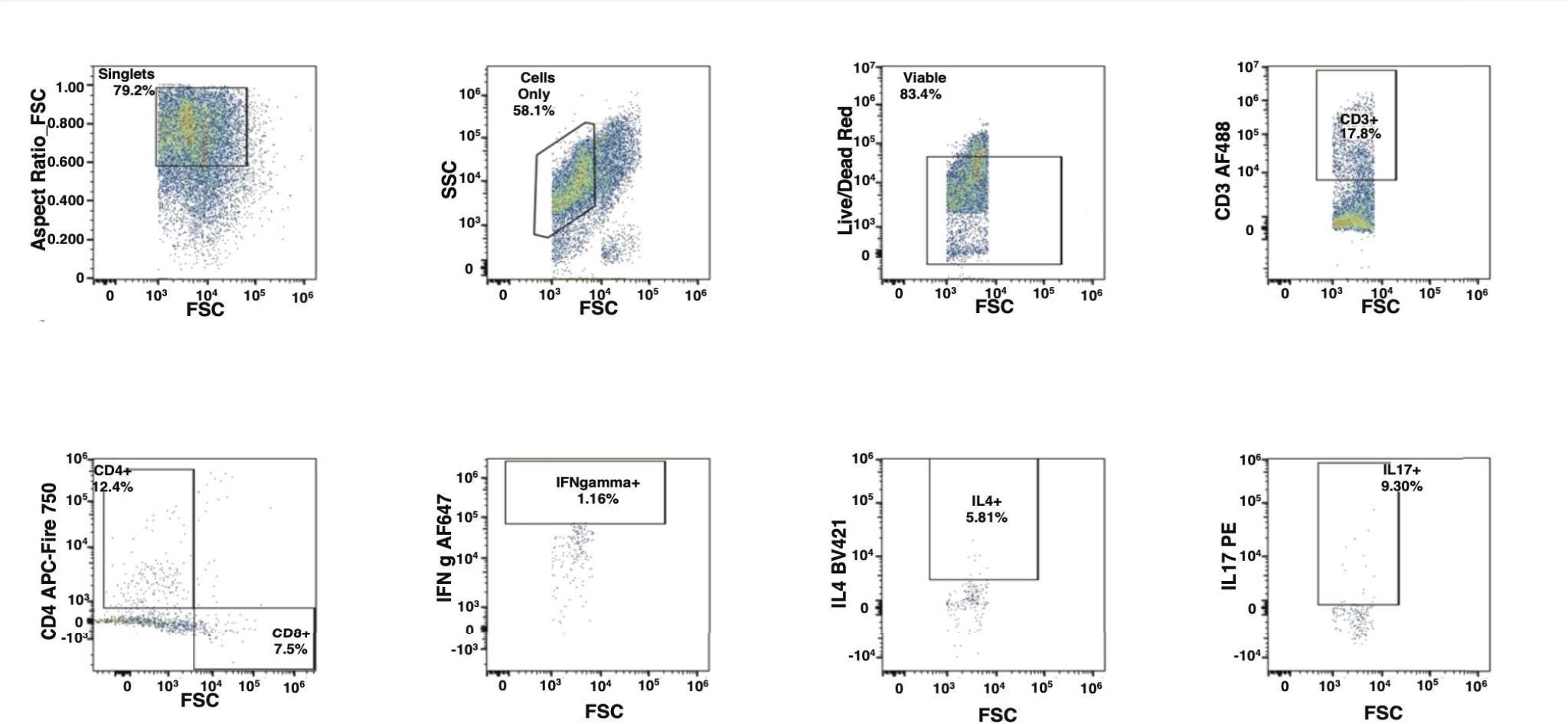


**Fig. S2**

**Flow cytometry gating strategy for Th1, Th2, Th17, and CD8+ populations, FA-HI sample.** Gating from left to right, top to bottom: Singlets, cells only, viable, CD3+ cells, CD4+ cells and CD8+ cells, IFN-γ, IL-4, and IL-17


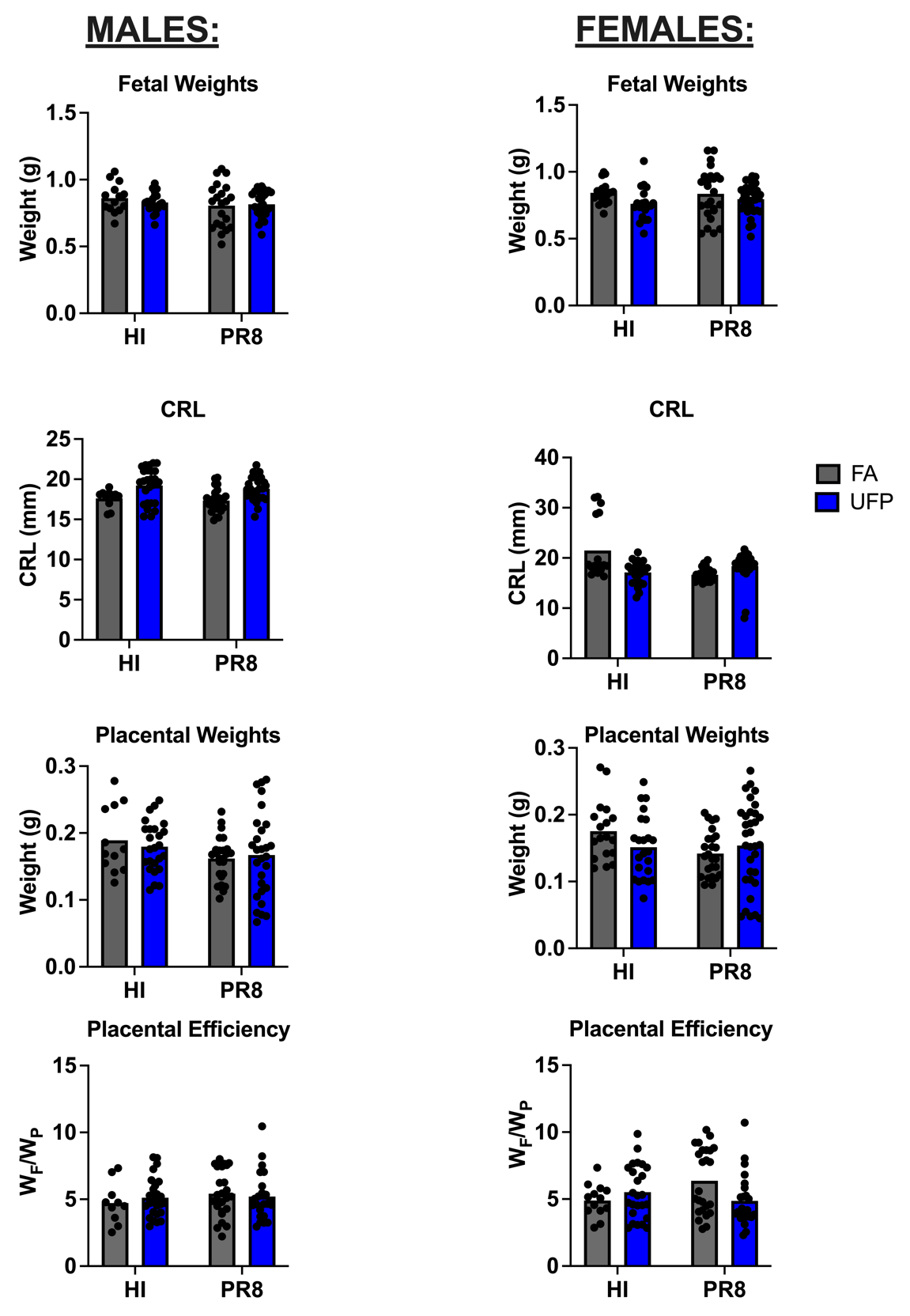


**Fig. S3**

**Fetal outcomes, stratified by sex.** Fetal weights, crown-to-rump lengths, placental weights, and placental efficiency (weight of fetus, W_F_, divided by weight of placenta, W_P_) for males (left) and females (right).

**
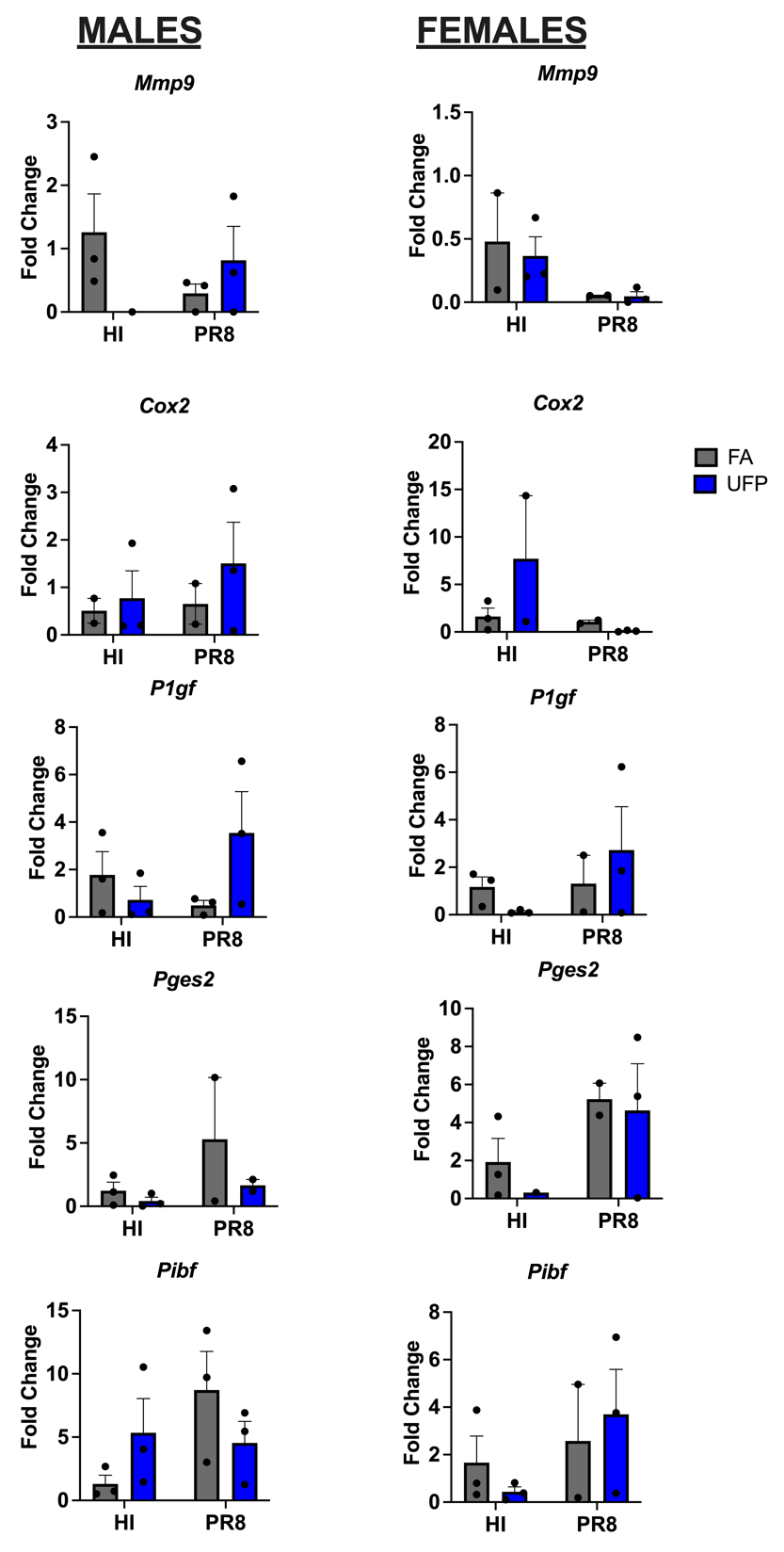
**

**Fig. S4**

**Placental qPCR, stratified by sex.** Fold change values for Mmp9, Cox2, P1gf, Pges2, and Pibf for males (left) and females (right). Placentas (n = 3/sex/exposure group) were pooled between litters.

| **Table S1: Lung qPCR primer sequences** | | | |
| --- | --- | --- | --- |
| **Gene** | **Accession Number** | **Sequence (5’ – 3’)** | **Amplicon Size** |
| *Gapd(h)* (housekeeping)  Forward  Reverse | NM_001289726.2 | TGTCAAGCTCATTTCCTGGTATGACA  GAGTTGGGATAGGGCCTCTCTT | 148 |
| *InfA*  Forward  Reverse | MT860791 | GGACTGCAGCGTAGACGCTT  CATCCTGTTGATATTGAGGCCCAT | 165 |
| *Tgf-*$\beta$  Forward  Reverse | NM_011577 | CAAGGGCTACCATGCCAACT  GTACTGTGTGTCCAGGCTCCAA | 167 |
| *Il-1* $\beta$  Forward  Reverse | NM_008361 | GCCACCTTTTGACAGTGATGAG  AAGGTCCACGGGAAAGACAC | 219 |
| *Il-33*  Forward  Reverse | NM_001164724 | TCCAACTCCAAGATTTCCCCG  CATGCAGTAGACATGGCAGAA | 120 |
| *Irf5*  Forward  Reverse | NM_001252382 | GTCTCTGGCCGCTGAGGTAT  CCTGGGGCTGAGTGGTTCAT | 187 |
| *Irf7*  Forward  Reverse | NM_016850 | CTCAGCAATGCTCTGCCCAC  AACACGGTCTTGCTCCTGGC | 207 |
| *Stat4*  Forward  Reverse | NM_001308266 | TCGTTCAAGCGTGTCCTGGC  AGCAGATGCCGGATTTCCATAG | 201 |
| *Sphk1*  Forward  Reverse | NM_025367 | GATTGGATCTGGCCGCCCC  TTCAGCAGCACCAGCACTCT | 262 |
| *Nos2*  Forward  Reverse | NM_010927 | GGTGAAGGGACTGAGCTGTTA  TGAAGAGAAACTTCCAGGGGCA | 163 |

| **Table S2: Placenta qPCR Primer Sequences** | | | |
| --- | --- | --- | --- |
| **Gene** | **Accession Number** | **Sequence (5’ – 3’)** | **Amplicon Size** |
| *Gapd(h)* (housekeeping)  Forward  Reverse | NM_001289726.2 | TGTCAAGCTCATTTCCTGGTATGACA  GAGTTGGGATAGGGCCTCTCTT | 148 |
| *Vegf*  Forward  Reverse | NM_001025250.3 | CAGACAGTGCTCCAGCCG  CTCTCCTTCTGTCGTGGGTGC | 260 |
| *P1gf*  Forward  Reverse | NM_008827.3 | CAGGTCCTAGCTGGGTTGGC  TCATCACCACAGCAGCCACT | 224 |
| *Cox-2*  Forward  Reverse | NM_011198.5 | ACATCCCCTTCCTGCGAAGT  GGGCAGTCATCTGCTACGGG | 169 |
| *Pibf*  Forward  Reverse | NM_029320.3 | TGGCACTGGATCTGGAGCAG  TCTTGGGCCACTCTTGTGCT | 240 |
| *Pges2*  Forward  Reverse | NM_133783.2 | ACAGCCGTGGGTAAAGACCG  GCTGGATGTGTGAGTGTCGC | 136 |
